# Supplementary material for: Active forest stewardship benefits priority birds in the New Jersey Pine Barrens
Source: PLoS One. 2024 Jun 20;19(6):e0302040. doi: 10.1371/journal.pone.0302040 (PMC11189188; doi:10.1371/journal.pone.0302040)
Supplement: S1 Table — Bolded values indicate those value above our cutoff of |r| = 0.7. (DOCX) [file pone.0302040.s001.docx]

S1 Table

|  | % Grass | % Forbes | % Woody | % Litter | % Bare ground | Basal density | Canopy coverage | VOR 0.25 | VOR 1 | VOR 2 |
| --- | --- | --- | --- | --- | --- | --- | --- | --- | --- | --- |
| % Grass | 1 | **0.72** | -0.28 | **-0.77** | **0.74** | -0.30 | -0.33 | -0.10 | -0.14 | -0.16 |
| % Forbes |  | 1 | **-0.71** | 0.14 | -0.14 | -0.01 | 0.20 | -0.01 | -0.14 | -0.07 |
| % Woody |  |  | 1 | **-0.74** | **-0.72** | 0.34 | 0.23 | 0.31 | 0.27 | 0.09 |
| % Litter |  |  |  | 1 | -0.01 | -0.22 | -0.08 | -0.13 | -0.09 | 0.11 |
| % Bare ground |  |  |  |  | 1 | -0.34 | -0.35 | -0.35 | -0.16 | -0.08 |
| Basal density |  |  |  |  |  | 1 | **0.88** | 0.21 | 0.16 | 0.10 |
| Canopy Coverage |  |  |  |  |  |  | 1 | 0.12 | -0.03 | -0.29 |
| VOR 0.25 |  |  |  |  |  |  |  | 1 | **0.77** | **0.74** |
| VOR 1 |  |  |  |  |  |  |  |  | 1 | **0.76** |
| VOR 2 |  |  |  |  |  |  |  |  |  | 1 |
