# Supplementary material for: Active forest stewardship benefits priority birds in the New Jersey Pine Barrens
Source: PLoS One. 2024 Jun 20;19(6):e0302040. doi: 10.1371/journal.pone.0302040 (PMC11189188; doi:10.1371/journal.pone.0302040)
Supplement: Attachment — Submitted filename: Reviewer2comments.docx [file pone.0302040.s002.docx]

**Reviewer 2 comments:**

**Line 49:** remove comma after forests

**Line 50:** give some brief examples of how/why thinning helps birds

**Line 57:** same as in line 50 – why?

**Line 69:** what does diminished state means?

**Line 79:** why mention mammals? If so, what is their response?

**Line 86:** why focusing on only the highest priority species? With a community model framework, why do you choose only 12 species instead of using the whole community to borrow statistical strength from more common species? Species of high concern are generally rare or find in smaller numbers, so using common species and sharing information would make results more robust.

**Line 89:** In an opposite thought of the previous comment, how do you think that grouping a small number of species of different ecologies in forest groups might be influencing your results? Do you expect an oriole to have a similar response to a warbler? Justify a little the choices in line 89 and 86 would help the reader understand your study a little more

**Line 98:** explain what community, suite and species levels are

**Line 105:** you can use the three terms the first time (Scrub-Shrub/Early Successional/Young Forest), nut choose only one hereafter in the text

**Line 168:** since the values sum to one, aren’t they highly correlated to each other?

**Line 172 and 177:** did you only measure canopy cover for non-grassland sites?

**Line 190:** It would be good to test and mention that your covariates are not correlated to each other

**Line 194:** lower case *i*

**Line 261:** can you justify a little why you chose such a large value of correlation threshold? 0.7 is a lot. I would remove variables that are so correlated with each other.

**Line 269:** you mention sampling birds at 0-50m and 50-100m radii. where was this used in the model? If not, remove from text.

**Line 274:** why (%)? Remove; as mentioned before, including variables that have percentages make the levels within the same variable very correlated to each other, e.g. if one variable is 40%, the other will be 60%, if one is 15%, the other will be 85%. I would check the correlation between the levels of the variable that you are including in the model. I would mention the variables that you removed from the model in the methods (when you mention the 0.7 threshold) and not have it again here. Because you mention this in the results, the reader has no idea of how many parameters you are actually using. Showing the vectors with all you environmental covariates in the methods section will help.

**Line 282:** r **<** 0.35, not greater

**Line 287**: a figure would be very helpful for the values in table 1

**Line 288:** I would mention the binning in the methods section, or not even mention it at all and just have the 0-100m radius in the text.

**Line 292:** include the traceplots and a table with the r-hat values.

**Line 304:** it is still very unclear what community is – is it all the 3 suites combined? If so, does it make sense to clump species with such different ecology and habitat choices, as well as opposite responses to vegetation variables in a group? You should clearly define what the bcr, community and suite levels are.

**Line 305:** show the histogram of the posteriors for the parameters. You can put both these and the traceplots in supplementary. Posterior predictive checks and pairs plots should be in supplementary too.

**Line 308:** because of the nature of your standardized coefficients, it is not clear what does a increase of 0.46 birds is. Can you put it in a scale of 1 or 10 birds for each x amount of basal area

**Line 343:** since you focused on high priority species, maybe you should say that your goal was to ensure their presence, and not the presence of the whole community. A more diverse community would include all species, and not focus on a subset.

**Line 343:** how does that result applies to grasslands? Could you please have a table with all the covariates used for each of the three suites of habitats? Or did you just put zero for the basal area in grasslands? Explain how you made the choice of putting a zero or removing the variable for one of the suite levels. Also, please explain in the methods how variables were measured for grasslands. You only have a species for grasslands. Given that, is it worth it to include it?

**Line 349:** but did the early successional and grassland species increased in the reduced basal area pine stands, or in the early successional and grasslands that have reduced basal area? If it was not in the pine stands, the diversity didn’t really increase with the thinning, only in the whole NJPB. I think you meant that the diversity increased in the grasslands and early successional with thinning on those areas – could you make it a little clearer in the text. Also mention in what habitat these results are true for in the 9 citation.

**Line 363:** why did species had such different responses? Were the group responses similar?

**Line 417:** autecological?

**Line 446:** are those recommendations for forest? Grasslands? Early succession? And please explain again where that number came from.

**Line 448:** same as before – include a citation for that recommendation, or briefly mention what result that you have that indicate this statement.

**Figure 2:** you can make the x-axis smaller (like panel 1 from -0.25 to 0.3) to highlight your results better and make the values clearer – they look a little small. I would also write the variable names on the axis titles and put the symbol from the equation (beta_grass) in parenthesis. I would also put the units or mention they were standardized.

**General:**

I would focus more on why the forest covariates that you choose are relevant for birds, and how they affect the different birds in the different habitat types. I understand that the NJPB is an important and super relevant conservation area for birds, but I would focus a little more in why the covariates are helping (or not) bird populations, rather than so much in how the distribution of those variables in NJPB is. I missed in the discussion some more ecological explanations why the 3 different groups of species respond the way they do to thinning – for example, how would a more open understory benefit a grassland species (food availability, nest site availability, etc), but that was not true for the forest species. This is super interesting research about different treatment types, but also about how and why species respond to them.

I would also explain well how you measured the different variables for the different habitat types (like basal area for forest versus grasslands), and if it makes sense to have a BCR response that includes these two types of responses, since they can have opposite signals within the groups, making the BCR estimate zero.

I would add figures with the effects of the covariates at the 3 groups and BCR level. Posterior density plots showing the results for species, and them for the groups would be very helpful.

It is a really good paper, with an amazing study design and impressive dataset.

Congrats and thanks for the opportunity to learn more about this system! It is a very interesting study and I look forward to see it published!
