# Supplementary material for: Active forest stewardship benefits priority birds in the New Jersey Pine Barrens
Source: PLoS One. 2024 Jun 20;19(6):e0302040. doi: 10.1371/journal.pone.0302040 (PMC11189188; doi:10.1371/journal.pone.0302040)
Supplement: Attachment — Submitted filename: PLOS ONE revision 2 reviewer responses.docx [file pone.0302040.s003.docx]

**Editor Comments:**
Line 49 - delete comma after "forests" (also noted by a reviewer)

*Response: Done*

Line 185 - delete "is plausible" (also noted by a reviewer)

*Response: Done*

Line 186 - replace "his" with "this"

*Response: Done*

Line 194 - "i" should be lower-case (also noted by a reviewer)

*Response: Done*

Lines 233-236 - this sentence could form part of your response to the reviewer's question (referencing line 86) about why you focused on 12 particular bird species in your study.

*Response: Thank you*

Line 260 - replace "or" with "of"

*Response: Done*

Lines 343-345 - This sentence reads a little awkwardly. I suggest inserting "whether" after "explore"

*Response: Done*

Line 417 - "autecological" seems like an odd word choice here. Why not just say "ecological needs"? (also noted by a reviewer)

*Response: Done*

Line 419 - delete extra space after "[71]"

*Response: Done*

Line 431 - delete extra space after "met"

*Response: Done*

Line 452 - "2020 NJ Forest Action Plan" - reading this again reminded me about a nagging question that I meant to pose during the previous review round. Your study deals with observations dating from 2017 or earlier. Has anything changed in the time since? It would make sense, of course, for the 2020 NJ Forest Action Plan to be based on observations and analyses from the previous decade. However, we're now seven years past the end of data collection for your study. Perhaps forest conditions, etc., haven't changed significantly in the NJPB over this time. On the other hand, it's also been a few years since the release of the Forest Action Plan. Can you comment on anything regarding implementation of the Forest Action Plan, or on any other aspects of the NJPB that may be different from several years ago?

*Response: Thanks for that question. The answer is nothing has changed. Politically, the system has been very slow (decades long call for a change in management) as well as a multiyear hiccup with covid shutting down most recent efforts. So we feel confident that the data and recommendations surrounding this paper are up-to-date.*

**Reviewer #2**
Line 191 (line 167 clean): What is the "ecological selection process"? What defines "habitat quality"? Please clarify this text.

*Response: We simplified the sentence to be more clear.*

Line 213 (line 185 clean): Consider dropping "is plausible" as you are not making the argument for plausibility here only that you included these as covariates.

*Response: Done*

Line 215 (line 186 clean): "his" should be 'this'

*Response: Done*

Lines 213-219 (lines 185-291 clean): This is an exceptionally long, and meandering sentence. I would consider breaking this sentence into several, each with a unique topic. As is, it is really difficult to understand what you are trying to convey in this sentence.

*Response: Done*

Line 267: Conducting surveys on a roadway seems an illogical condition for not requiring IACUC, or related, approval. Perhaps this is the case but I think this would be questioned by a reader who might be familiar with, or associated with an institution that has different requirements towards IACUC protocols.

*Response: True and we have clarified sentence.*

Line 397 (line 345 clean): Was your goal to explore 'if'...?

*Response: Done*

Line 405 (line 352 clean): What does improving abundance and biodiversity mean? In some instances, a decrease in biodiversity through loss of non-native, negatively-affecting species would be beneficial, others situations a gain in biodiversity would be best. 'Improve' is a vague term here that needs to be reformed with specificity.

*Response: We apologize, the correct word was “increase”*

Line 431 (line 376 clean): Please say with additional detail what you mean with "more refined temporal evaluations"

*Response: Ultimately, we decided to delete the sentence as it was overly informative or helpful.*

Literature cited: Please check references for consistent formatting. Some references have spaces before page numbers, others not. you also have some journals listed with different names (eg. "The Auk" vs. "Auk").

*Response: Done*

Table 2: Include "parameters" in last sentence of heading as covariates, parameters, and suites are all noted in this table.

*Response: Done*

**Reviewer 2 (or is it 3?) comments:**

**Line 49:** remove comma after forests

*Response: Done*

**Line 50:** give some brief examples of how/why thinning helps birds

*Response: Done*

**Line 57:** same as in line 50 – why?

*Response: Done*

**Line 69:** what does diminished state means?

*Response: Removed*

**Line 79:** why mention mammals? If so, what is their response?

*Response: Clarified*

**Line 86:** why focusing on only the highest priority species? With a community model framework, why do you choose only 12 species instead of using the whole community to borrow statistical strength from more common species? Species of high concern are generally rare or find in smaller numbers, so using common species and sharing information would make results more robust.

*Response: Our focus was on the predetermined priority conservation species as defined by the U.S. Fish and Wildlife Service’s Atlantic Coast Joint Venture and Mid-Atlantic and New England Coastal Bird Conservation Region 30 Implementation Plan. Additionally, we restricted our modeling effort to the 12 priority species which did not include nomadic and aerial coursing species (e.g., raptors), which may have home ranges that are orders of magnitude larger than those species targeted here.*

**Line 89:** In an opposite thought of the previous comment, how do you think that grouping a small number of species of different ecologies in forest groups might be influencing your results? Do you expect an oriole to have a similar response to a warbler? Justify a little the choices in line 89 and 86 would help the reader understand your study a little more.

*Response: We have attempted to clarify these sentences.*

**Line 98:** explain what community, suite and species levels are.

*Response: Done.*

**Line 105:** you can use the three terms the first time (Scrub-Shrub/Early Successional/Young Forest), but choose only one hereafter in the text

Response: Done and we chose “Scrub-Shrub”

**Line 168:** since the values sum to one, aren’t they highly correlated to each other?

*Response: Thank you for that question. While it is possible an individual point could have correlated values of percent ground cover since they sum to one, this is less of an issue across the diversity of site point collections. This is a standard assumption for Daubenmire frame microscale habitat collection. That being said, we are sensitive to correlation and we standardized all continuous covariates, centering each on a mean of 0 and standard deviation of 1, to improve model convergence and interpretability. We used Pearson’s correlation tests to assess the degree of collinearity of model parameters and did not fit models when* *|r| > 0.7. This fact is later explained in the methods. And then we talk about removal of correlated variables in the results.*

**Line 172 and 177:** did you only measure canopy cover for non-grassland sites?

*Response: We collected at all site but of course the answer was always “no canopy cover” in early successional sites.*

**Line 190:** It would be good to test and mention that your covariates are not correlated to each other

*Response: We apologize, we do not understand this comment in relation to Line 190. However, we think our discussions of the correlation topics in the later paragraphs should address this comment.*

**Line 194:** lower case *i*

*Response: Done*

**Line 261:** can you justify a little why you chose such a large value of correlation threshold? 0.7 is a lot. I would remove variables that are so correlated with each other.

*Response: The range of correlation differs across studies but when dealing with datasets with large sample sizes (as we had) the use of 0.7 is robust to still detect biologically significant results hence our use of this value.*

**Line 269:** you mention sampling birds at 0-50m and 50-100m radii. where was this used in the model? If not, remove from text.

*Response: I’m sorry, we do not have reference to this in the text so we believe this is already done.*

**Line 274:** why (%)? Remove; as mentioned before, including variables that have percentages make the levels within the same variable very correlated to each other, e.g. if one variable is 40%, the other will be 60%, if one is 15%, the other will be 85%. I would check the correlation between the levels of the variable that you are including in the model. I would mention the variables that you removed from the model in the methods (when you mention the 0.7 threshold) and not have it again here. Because you mention this in the results, the reader has no idea of how many parameters you are actually using. Showing the vectors with all your environmental covariates in the methods section will help.

*Response: I suppose the issue is whether to include the correlation results in the Methods or the* *Results. We thought about this request and ultimately decided to keep it in the results. We felt it was more important to talk about the wide range of variables that we collected in the field in the Methods and then present the pairing of the data (due to correlations) for our model building as part of our analysis/results. Taking out the first 2/3rds of the first paragraph of the results to be inserted in the Methods made the presentation of the remaining sentences of the first paragraph of the Results confusing.*

**Line 282:** r **<** 0.35, not greater

*Response: Done*

**Line 287**: a figure would be very helpful for the values in table 1

*Response: We appreciate this recommendation, however after considering the wide range of the values, we ultimately decided it would be hard to see the differences in the box and whisker plot and decided to stick with the specificity of numbers in a table. We apologize.*

**Line 288:** I would mention the binning in the methods section, or not even mention it at all and just have the 0-100m radius in the text.

*Response: I’m sorry, there is no mention of 0-50 and 51-100 binning in the current version of the text so we believe this has been resolved.*

**Line 292:** include the traceplots and a table with the r-hat values.

*Response: We have added a table of the correlations to supplement the first paragraph of results.*

**Line 304:** it is still very unclear what community is – is it all the 3 suites combined? If so, does it make sense to clump species with such different ecology and habitat choices, as well as opposite responses to vegetation variables in a group? You should clearly define what the bcr, community and suite levels are.

*Response: On line 30 and 101 we explain what community at the Bird Conservation Region 30 and suite habitats are. While we primarily interested in the suite level habitats since birds are evolved to those, we still felt it was important to look at the pine barrens as a whole to address ecosystem habitat management impacts of the birds of conservation concern.*

**Line 305:** show the histogram of the posteriors for the parameters. You can put both these and the traceplots in supplementary. Posterior predictive checks and pairs plots should be in supplementary too.

*Response: We have included a supplemental table of pairwise correlations of habitat variables but decided to keep Figure 2 and Figure 3 in the main body of the text.*

**Line 308:** because of the nature of your standardized coefficients, it is not clear what does a increase of 0.46 birds is. Can you put it in a scale of 1 or 10 birds for each x amount of basal area

*Response: Done*

**Line 343:** since you focused on high priority species, maybe you should say that your goal was to ensure their presence, and not the presence of the whole community. A more diverse community would include all species, and not focus on a subset.

*Response: Done*

**Line 343:** how does that result applies to grasslands? Could you please have a table with all the covariates used for each of the three suites of habitats? Or did you just put zero for the basal area in grasslands? Explain how you made the choice of putting a zero or removing the variable for one of the suite levels. Also, please explain in the methods how variables were measured for grasslands. You only have a species for grasslands. Given that, is it worth it to include it?

*Response: There are no true grasslands prairies in the New Jersey Pine Barrens but we can have grassland dominant species occurring in open managed pine savannah settings. Therefore, all microhabitat measurements are plausible with no guaranteed zeros.*

**Line 349:** but did the early successional and grassland species increased in the reduced basal area pine stands, or in the early successional and grasslands that have reduced basal area? If it was not in the pine stands, the diversity didn’t really increase with the thinning, only in the whole NJPB. I think you meant that the diversity increased in the grasslands and early successional with thinning on those areas – could you make it a little clearer in the text. Also mention in what habitat these results are true for in the 9 citation.

*Response: This relates to the last questions and response. There are no grasslands in the pine barrens and so we are discussing reducing basal density of the pine savannah system. For these comments we added a few words to hopefully make it clearer.*

**Line 363:** why did species had such different responses? Were the group responses similar?

*Response: This is just a function of certain variations that occur in niche portioning across species even if general preference for certain types of ecosystems.*

*We have added a clarification.*

**Line 417:** autecological?

*Response: Fixed*

**Line 446:** are those recommendations for forest? Grasslands? Early succession? And please explain again where that number came from.

*Response: As mentioned before there are no grasslands and this is just a pine forest or pine-savannah system but we have clarified the sentence.*

**Line 448:** same as before – include a citation for that recommendation, or briefly mention what result that you have that indicate this statement.

*Response: Done*

**Figure 2:** you can make the x-axis smaller (like panel 1 from -0.25 to 0.3) to highlight your results better and make the values clearer – they look a little small. I would also write the variable names on the axis titles and put the symbol from the equation (beta_grass) in parenthesis. I would also put the units or mention they were standardized.

*Response: We are terribly sorry but our statistician who made this graph had a personal emergency since the first submission of the manuscript and left the project with no forwarding contact information. We are now not able to make a new graph given the limited files he left us with.*

**General:**

I would focus more on why the forest covariates that you choose are relevant for birds, and how they affect the different birds in the different habitat types. I understand that the NJPB is an important and super relevant conservation area for birds, but I would focus a little more in why the covariates are helping (or not) bird populations, rather than so much in how the distribution of those variables in NJPB is. I missed in the discussion some more ecological explanations why the 3 different groups of species respond the way they do to thinning – for example, how would a more open understory benefit a grassland species (food availability, nest site availability, etc), but that was not true for the forest species. This is super interesting research about different treatment types, but also about how and why species respond to them.

*Response: With the greatest of respect, we feel like our existing discussions does in fact provide numerous examples that discuss this topic, however we have added a few clarifying sentences throughout.*

I would also explain well how you measured the different variables for the different habitat types (like basal area for forest versus grasslands), and if it makes sense to have a BCR response that includes these two types of responses, since they can have opposite signals within the groups, making the BCR estimate zero.

*Response: As mentioned previously, we are working in a pine and pine savannah system and all vegetation measurements were plausible across the landscape.*

I would add figures with the effects of the covariates at the 3 groups and BCR level. Posterior density plots showing the results for species, and them for the groups would be very helpful.

It is a really good paper, with an amazing study design and impressive dataset.

*Response: Unfortunately, our problem with the lost statistician has put us in a tough bind for creating new figures. We apologize we cannot accomplish this request.*

Congrats and thanks for the opportunity to learn more about this system! It is a very interesting study and I look forward to see it published!

*Response: Thank you!*
